# Oxidative Stress and Cardiovascular Complications in Type 2 Diabetes: From Pathophysiology to Lifestyle Modifications

**DOI:** 10.3390/antiox14010072

**Published:** 2025-01-09

**Authors:** Alfredo Caturano, Maria Rocco, Giuseppina Tagliaferri, Alessia Piacevole, Davide Nilo, Giovanni Di Lorenzo, Ilaria Iadicicco, Mariarosaria Donnarumma, Raffaele Galiero, Carlo Acierno, Celestino Sardu, Vincenzo Russo, Erica Vetrano, Caterina Conte, Raffaele Marfella, Luca Rinaldi, Ferdinando Carlo Sasso

**Affiliations:** 1 Department of Advanced Medical and Surgical Sciences, University of Campania Luigi Vanvitelli, 80138 Naples, Italy; alfredo.caturano@unicampania.it (A.C.); maria.rocco@studenti.unicampania.it (M.R.); giuseppina.tagliaferri@gmail.com (G.T.); alessia0694@hotmail.it (A.P.); nilodavide@gmail.com (D.N.); giuann86@gmail.com (G.D.L.); ilariaiad@gmail.com (I.I.); mariarosaria.donnarumma@unicampania.it (M.D.); raffaele.galiero@unicampania.it (R.G.); celestino.sardu@unicampania.it (C.S.); erica.vetrano@unicampania.it (E.V.); raffaele.marfella@unicampania.it (R.M.); 2 Department of Human Sciences and Promotion of the Quality of Life, San Raffaele Roma Open University, 00166 Rome, Italy; caterina.conte@uniroma5.it; 3 Azienda Ospedaliera Regionale San Carlo, 85100 Potenza, Italy; carlo894@gmail.com; 4 Sbarro Institute for Cancer Research and Molecular Medicine, Center for Biotechnology, Department of Biology, College of Science and Technology, Temple University, Philadelphia, PA 19122, USA; v.p.russo@libero.it; 5 Division of Cardiology, Department of Medical Translational Sciences, University of Campania Luigi Vanvitelli, 80138 Naples, Italy; 6 Department of Endocrinology, Nutrition and Metabolic Diseases, IRCCS MultiMedica, 20099 Milan, Italy; 7 Department of Medicine and Health Sciences “Vincenzo Tiberio”, Università degli Studi del Molise, 86100 Campobasso, Italy

**Keywords:** oxidative stress, type 2 diabetes mellitus, cardiovascular complications, lifestyle interventions, Mediterranean diet, pathophysiology

## Abstract

Type 2 diabetes mellitus (T2DM) is a chronic metabolic disorder that significantly increases the risk of cardiovascular disease, which is the leading cause of morbidity and mortality among diabetic patients. A central pathophysiological mechanism linking T2DM to cardiovascular complications is oxidative stress, defined as an imbalance between reactive oxygen species (ROS) production and the body’s antioxidant defenses. Hyperglycemia in T2DM promotes oxidative stress through various pathways, including the formation of advanced glycation end products, the activation of protein kinase C, mitochondrial dysfunction, and the polyol pathway. These processes enhance ROS generation, leading to endothelial dysfunction, vascular inflammation, and the exacerbation of cardiovascular damage. Additionally, oxidative stress disrupts nitric oxide signaling, impairing vasodilation and promoting vasoconstriction, which contributes to vascular complications. This review explores the molecular mechanisms by which oxidative stress contributes to the pathogenesis of cardiovascular disease in T2DM. It also examines the potential of lifestyle modifications, such as dietary changes and physical activity, in reducing oxidative stress and mitigating cardiovascular risks in this high-risk population. Understanding these mechanisms is critical for developing targeted therapeutic strategies to improve cardiovascular outcomes in diabetic patients.

## 1. Introduction

Type 2 diabetes mellitus (T2DM) is a long-standing metabolic disorder defined by insulin resistance, impaired glucose metabolism, and the gradual loss of beta-cell function [1]. In recent decades, its global prevalence has surged significantly, largely due to rising obesity rates, sedentary behaviors, and aging populations [2,3]. Currently, more than 537 million adults worldwide are living with diabetes, with T2DM making up around 90% of these cases, according to the International Diabetes Federation [4]. Beyond placing a substantial burden on healthcare systems, T2DM greatly affects patients’ quality of life due to the wide range of chronic complications it entails [5,6]. Among the long-term complications of T2DM, cardiovascular disease (CVD) stands out as the leading cause of both morbidity and mortality [7,8]. Individuals with T2DM are at a two- to four-fold greater risk of developing cardiovascular complications compared to non-diabetic individuals [9]. These complications encompass a range of conditions, including atherosclerosis, myocardial infarction, heart failure, diabetic cardiomyopathy, and stroke, all of which significantly contribute to reduced life expectancy in diabetic patients [10,11,12]. The strong association between T2DM and CVD underscores the urgent need to unravel the underlying mechanisms driving this relationship, with oxidative stress emerging as a key player [11,13]. Oxidative stress has emerged as a central pathophysiological factor in both the development and progression of T2DM-related cardiovascular complications [14]. Defined as an imbalance between the production of reactive oxygen species (ROS) and the body’s ability to detoxify these reactive intermediates or repair the resulting damage, oxidative stress is now recognized as a key driver of cellular injury in diabetic patients [15,16]. This state of heightened oxidative burden leads to damage in lipids, proteins, and DNA, contributing to endothelial dysfunction, vascular inflammation, and subsequent cardiovascular events [17,18,19].

In the context of T2DM, chronic hyperglycemia plays a pivotal role in exacerbating oxidative stress [14]. Elevated blood glucose levels stimulate multiple pathways that enhance ROS production, including the formation of advanced glycation end products (AGEs), the activation of the polyol pathway, and mitochondrial dysfunction [15]. These processes generate excessive ROS that overwhelm the body’s antioxidant defenses, setting the stage for widespread tissue damage [14,20]. Additionally, oxidative stress perpetuates a vicious cycle of inflammation and endothelial damage, which is particularly detrimental to vascular health in diabetic patients [17,21].

Given the critical role of oxidative stress in the pathophysiology of cardiovascular complications in T2DM, this review aims to provide an in-depth analysis of the molecular mechanisms by which oxidative stress contributes to CVD. Moreover, this review will examine how lifestyle modifications, including dietary changes and regular physical activity, can serve as effective strategies to reduce oxidative stress and mitigate cardiovascular risks in this vulnerable population.

## 2. Pathophysiology of Oxidative Stress in Type 2 Diabetes Mellitus

### 2.1. Hyperglycemia-Induced Oxidative Stress

**Figure 1:**
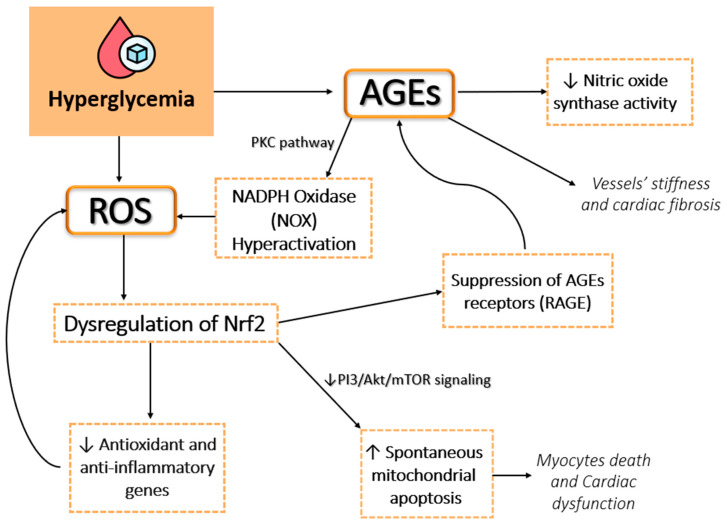
This figure illustrates the cascading effects of chronic hyperglycemia on cellular and molecular pathways, leading to oxidative stress, advanced glycation end products (AGEs) formation, and cardiovascular damage. Hyperglycemia induces the overproduction of reactive oxygen species (ROS) through NADPH oxidase (NOX) hyperactivation, facilitated by the protein kinase C (PKC) pathway. ROS accumulation dysregulates the NRF2 pathway, reducing the expression of antioxidant and anti-inflammatory genes, thereby exacerbating oxidative damage. Concurrently, hyperglycemia accelerates the formation of AGEs, which suppress AGE receptor (RAGE) signaling and nitric oxide synthase activity. These changes contribute to vessel stiffness, cardiac fibrosis, and impaired endothelial function. Additionally, the suppression of the PI3K/Akt/mTOR signaling cascade leads to increased spontaneous mitochondrial apoptosis, resulting in cardiomyocyte death and cardiac dysfunction.

Hyperglycemia is a central contributor to tissue damage and organ dysfunction in T2DM, largely through the induction of oxidative stress [22]. Chronic high blood glucose levels promote excessive ROS production, leading to inflammation, cellular damage, and complications, particularly in the cardiovascular and renal systems [22]. Several key mechanisms are involved in this process, including the polyol pathway, AGEs, protein kinase C (PKC) activation, and the hexosamine pathway [23]. These interconnected pathways collectively elevate ROS production, perpetuate inflammation, and contribute to the progression of diabetic complications (Figure 1) [14].

One of the most significant mechanisms is PKC activation, a critical pathway contributing to hyperglycemia-induced oxidative stress [24]. Hyperglycemia leads to increased diacylglycerol (DAG) levels, a primary activator of PKC. Additionally, PKC activation is induced by oxidative stress, angiotensin II, platelet-derived growth factor, and vascular endothelial growth factor (VEGF), further amplifying its detrimental effects [25]. This activation triggers a cascade of detrimental effects on vascular function and tissue integrity [26]. PKC activation enhances ROS production by stimulating NADPH oxidase activity and impairing mitochondrial function. It also promotes vascular permeability and inflammation through the upregulation of VEGF and pro-inflammatory cytokines [27]. Furthermore, PKC activation exacerbates endothelial dysfunction by inhibiting endothelial nitric oxide synthase (eNOS) and reducing nitric oxide (NO) bioavailability [28]. PKC also disrupts blood flow regulation by increasing endothelin-1 (ET-1) expression, a potent vasoconstrictor [29]. Collectively, these processes amplify oxidative stress, inflammation, and vascular complications in diabetic tissues, linking PKC activation to the progression of microvascular and macrovascular complications in diabetes [30].

Under hyperglycemic conditions, mitochondrial dysfunction and oxidative stress are closely linked. Disruptions in glucose metabolism, such as increased aerobic glycolysis and altered glucose flux through the pentose phosphate pathway, promote ROS generation and mitochondrial dysfunction [24]. The polyol pathway, a major route for glucose metabolism under hyperglycemia, contributes to oxidative stress by consuming NADPH, reducing the antioxidant defense systems, and generating ROS in key tissues such as the heart, kidneys, retina, and vasculature [31,32]. Similarly, the hexosamine biosynthetic pathway (HBP) exacerbates oxidative stress and insulin resistance through protein modification by O-linked glycosylation [33]. Approximately 2–5% of intracellular glucose is diverted into HBP, where fructose-6-phosphate, a glycolytic intermediate, is converted into glucosamine-6-phosphate by the rate-limiting enzyme glutamine:fructose-6-phosphate amidotransferase (GFAT), using glutamine as a nitrogen donor [34]. This pathway culminates in the production of UDP-N-acetylglucosamine (UDP-GlcNAc), a substrate for O-GlcNAcylation. The enzyme, O-GlcNAc transferase (OGT), attaches O-GlcNAc to serine or threonine residues on proteins, a modification that is reversible by O-GlcNAcase (OGA) [35]. Under hyperglycemia, elevated glucose flux through the HBP increases UDP-GlcNAc levels, resulting in the excessive O-GlcNAcylation of proteins such as nuclear factor kappa B (NF-κB) and c-Jun N-terminal kinase (JNK) [36]. These modifications amplify oxidative stress by activating stress response pathways, disrupting mitochondrial function, and promoting ROS generation [37]. Together, these biochemical changes contribute to tissue-specific oxidative stress and low-grade inflammation, driving the pathological alterations seen in diabetes. Key transcription factors, such as NFκB, play a central role in this process, regulating the expression of pro-inflammatory cytokines, adhesion molecules, and enzymes that perpetuate oxidative damage and vascular dysfunction [38].

### 2.2. Role of Advanced Glycation End Products and the Polyol Pathway

AGEs are a major source of oxidative stress in T2DM, formed by the nonenzymatic glycation of proteins, lipids, and nucleic acids. This process accelerates in the presence of elevated blood glucose levels, contributing to tissue damage and the progression of diabetic complications [39,40]. AGEs alter cellular functions by modifying intracellular proteins, disrupting the extracellular matrix, and forming cross-links with vital molecules such as collagen and elastin, impairing tissue integrity and function [41,42,43]. AGE formation also leads to the activation of the receptor for AGE (RAGE), which further amplifies oxidative stress and inflammation by activating signaling pathways like NFκB and NADPH oxidase (NOX), perpetuating a cycle of damage and dysfunction in the vasculature and other tissues [44,45].

Under hyperglycemia, the polyol pathway becomes a significant contributor to oxidative stress by converting excess glucose into sorbitol and fructose. The enzyme aldose reductase catalyzes the reduction of glucose to sorbitol, utilizing NADPH as a cofactor. This consumption of NADPH depletes cellular reserves necessary for regenerating reduced glutathione, a critical antioxidant, thereby weakening the defense against ROS. Sorbitol is subsequently oxidized to fructose by sorbitol dehydrogenase, a reaction that uses NAD^+^ and further contributes to metabolic imbalance [46,47,48]. The accumulation of sorbitol increases osmotic stress within cells, while fructose undergoes glycation reactions that generate AGEs, amplifying oxidative damage and inflammation. These processes collectively exacerbate tissue injury, particularly in organs such as the heart, kidneys, and vasculature, where hyperglycemia-driven oxidative stress plays a pivotal role in diabetic complications [46,47,48,49].

### 2.3. Mitochondrial Dysfunction

Mitochondria are central to cellular energy production and ROS regulation. In T2DM, mitochondrial dysfunction is a key feature that exacerbates oxidative stress and impairs cellular metabolism [50,51]. Hyperglycemia drives mitochondrial dysfunction through multiple interconnected mechanisms, including excessive substrate influx into the electron transport chain (ETC), leading to electron leakage and the formation of superoxide radicals [24]. This overproduction of ROS overwhelms the cellular antioxidant defenses and damages mitochondrial DNA, proteins, and lipids, further impairing mitochondrial function [52,53].

A major contributor to mitochondrial dysfunction in hyperglycemia is the activation of metabolic pathways, such as the polyol and hexosamine biosynthetic pathways, which increase intracellular stress and disrupt mitochondrial homeostasis [54]. Elevated glucose flux through glycolysis generates excessive pyruvate, fueling the tricarboxylic acid cycle and increasing NADH and FADH_2_ supply to the ETC [55]. This heightened ETC activity promotes a hyperpolarized mitochondrial membrane potential, which accelerates ROS production [56,57]. Simultaneously, mitochondrial permeability transition pore (mPTP) opening, triggered by oxidative stress and calcium dysregulation, leads to the release of pro-apoptotic factors and further mitochondrial damage [58,59,60].

Mitochondrial dysfunction is closely linked to insulin resistance, as impaired oxidative phosphorylation reduces ATP production and exacerbates metabolic stress [58,59,60]. Hyperglycemia-induced mitochondrial morphological changes, such as swelling and fragmentation, serve as upstream drivers of excessive ROS production. While the exact mechanisms remain unclear, these alterations amplify metabolic stress and contribute to diabetic pathophysiology [50,61].

The impact of mitochondrial dysfunction extends to cardiovascular health, particularly in the myocardium and vasculature [62]. Excessive ROS production in the diabetic heart disrupts calcium handling, induces mPTP opening, and promotes apoptosis, which are central processes in the development of diabetic cardiomyopathy and heart failure [63,64,65]. In the vasculature, mitochondrial-derived ROS impair endothelial function, enhance vascular inflammation, and contribute to atherogenesis, further driving cardiovascular complications in diabetes [66].

### 2.4. Implications for Vascular Damage and Cardiovascular Disease

Oxidative stress, driven by mitochondrial dysfunction and ROS accumulation, is a critical factor in the pathogenesis of CVD in T2DM [63,64,65]. In diabetic tissues, including the myocardium and vasculature, excessive ROS production disrupts cellular homeostasis, impairs NO signaling, and induces endothelial dysfunction [67]. ROS reduces NO bioavailability by enhancing its degradation and impairing its synthesis, leading to vasoconstriction and inflammation [68,69]. Moreover, ROS-induced oxidative stress activates mitochondrial fission proteins such as Drp1 and Fis1, which further exacerbate mitochondrial dysfunction and ROS production in the vasculature. This vicious cycle promotes vascular inflammation, endothelial injury, and the progression of atherosclerosis in diabetic patients [67].

### 2.5. Endothelial Dysfunction and Vascular Inflammation

Endothelial dysfunction, a hallmark of diabetes, results from an imbalance between vasodilatory and vasoconstrictive factors. Oxidative stress diminishes the bioavailability of NO, a critical vasodilator, by enhancing its degradation and inhibiting its synthesis. The oxidation of tetrahydrobiopterin (BH4), an essential cofactor for endothelial nitric oxide synthase (eNOS), leads to eNOS uncoupling, further reducing NO levels and increasing superoxide production. This disruption in NO signaling contributes to endothelial dysfunction and vascular complications in T2DM [70,71,72]. In parallel, oxidative stress triggers inflammatory responses in the endothelium by activating transcription factors such as NFκB. This activation drives the expression of adhesion molecules (e.g., ICAM-1, VCAM-1), pro-inflammatory cytokines (e.g., MCP-1), and pro-thrombotic factors (e.g., PAI-1), creating a pro-inflammatory and pro-coagulant microenvironment. These changes promote leukocyte recruitment, increased vascular permeability, and endothelial cell injury, exacerbating vascular inflammation and dysfunction [73,74,75,76]. Additionally, reduced NO levels impair endothelial barrier integrity, facilitating the extravasation of inflammatory cells and lipoproteins into the subendothelial space, where they contribute to plaque formation [77]. Hyperglycemia-induced endothelial oxidative stress also upregulates ET-1, a potent vasoconstrictor, further skewing the balance toward vascular dysfunction [78]. Persistent endothelial injury and inflammation lead to vascular remodeling, increased arterial stiffness, and impaired angiogenesis, which are key contributors to the progression of diabetic vascular complications [79].

### 2.6. Role of Lipids in Oxidative Stress and Cardiovascular Risk

While glucose metabolism is central to the induction of oxidative stress in T2DM, lipids are equally significant contributors, particularly in the context of obesity, which is highly prevalent among patients with diabetes and strongly associated with CVD [80,81,82]. Dyslipidemia, often characterized by elevated triglycerides, low-density lipoprotein cholesterol (LDL), and reduced high-density lipoprotein cholesterol (HDL), exacerbates oxidative stress and inflammation in diabetic tissues [83,84]. One critical mechanism linking dyslipidemia to vascular injury is lipid peroxidation, which results in the accumulation of oxidized LDL (oxLDL) [85]. This modified lipoprotein activates endothelial cells, driving the expression of adhesion molecules such as ICAM-1 and VCAM-1 and pro-inflammatory cytokines that perpetuate vascular inflammation [86]. The interaction of oxLDL with scavenger receptors on macrophages further promotes foam cell formation and atherogenesis, processes central to the development of CVD [86].

Obesity-associated adipose tissue dysfunction further amplifies systemic oxidative stress through the increased secretion of free fatty acids (FFAs) and pro-inflammatory adipokines, including tumor necrosis factor-alpha and interleukin-6 [87]. These adipokines impair insulin signaling, enhance ROS production, and sustain inflammatory processes in vascular and myocardial tissues [88]. Elevated circulating FFAs, commonly observed in insulin-resistant states, undergo heightened β-oxidation, which, when dysregulated, leads to increased mitochondrial ROS generation [89]. This lipid-driven oxidative stress synergizes with hyperglycemia-induced mechanisms, compounding endothelial dysfunction, vascular inflammation, and CVD risk in diabetes [24]. The intertwined effects of dyslipidemia and glucose dysregulation underscore the multifaceted nature of oxidative stress in T2DM and its pivotal role in mediating vascular complications.

### 2.7. Genetic Factors Modulating Oxidative Stress Pathways

Genetic variations significantly influence the pathways driving oxidative stress and its downstream complications in T2DM, shaping the susceptibility to and progression of diabetic vascular disease [90]. Polymorphisms in genes encoding critical enzymes and regulatory proteins can alter their expression or activity, thereby modulating the effects of oxidative stress [90]. For example, variations in the aldose reductase gene, AKR1B1, impact polyol pathway activity, which has been implicated in the pathogenesis of diabetic nephropathy and retinopathy [91,92]. Similarly, polymorphisms in the PKC-β gene, PRKCB1, affect the activation of protein kinase C, a key player in oxidative stress and vascular inflammation, thereby influencing vascular outcomes in diabetes [93].

Genetic variants in NADPH oxidase subunits, such as NOX4, further contribute to increased ROS production, exacerbating endothelial dysfunction and enhancing the risk of vascular complications [94]. Additionally, polymorphisms affecting the regulation of nuclear factor-kappa B (NF-κB) influence the activation of this transcription factor and the subsequent expression of pro-inflammatory cytokines, contributing to variations in the severity of vascular inflammation observed among diabetic individuals [95]. These genetic factors interact with environmental triggers such as hyperglycemia and dyslipidemia, adding to the complexity of oxidative stress regulation. Insights into these genetic influences provide a foundation for developing personalized therapeutic strategies that target specific pathways in genetically predisposed individuals, thereby addressing the variability in the clinical presentation and progression of diabetic complications.

## 3. Molecular Mechanisms Linking Oxidative Stress to Cardiovascular Disease in Type 2 Diabetes Mellitus

ROS plays a pivotal role in the development of CVD in T2DM by promoting oxidative damage to endothelial cells, myocardiocytes, and vascular smooth muscle cells [96,97]. This oxidative damage leads to the activation of several pro-inflammatory and pro-thrombotic pathways, driving atherosclerosis, endothelial dysfunction, and vascular inflammation [96,98]. In physiological conditions, NOX plays a crucial role in supporting immune responses by generating ROS as part of the body’s defense mechanisms. However, in T2DM, NOX activity becomes dysregulated, particularly in fibroblasts, endothelial cells, and smooth muscle cells. This dysregulation leads to excessive ROS production, which contributes to chronic inflammation, endothelial dysfunction, and tissue damage, exacerbating the complications of T2DM [99,100]. NOX-generated superoxide ions play a critical role in LDL oxidation, plaque formation, and the progression of atherosclerosis. In diabetic animal models, elevated levels of NOX isoforms such as NOX2, NOX4, and NOX5 are observed in cells surrounding atheromatous plaques, driving oxidative stress and contributing to vascular inflammation. The inhibition or deletion of specific NOX isoforms, such as NOX2 and NOX4, significantly reduces atherosclerotic plaque formation and vascular inflammation, underscoring the importance of NOX in mediating cardiovascular complications in T2DM [101,102]. Additionally, NOX4 contributes to metabolic syndrome and vascular dysfunction by influencing adipocyte differentiation and vascular cell migration, further emphasizing the link between NOX activity and the pathophysiology of T2DM-associated CVD [101,102].

One critical mechanism linking oxidative stress to CVD in T2DM involves the nuclear factor erythroid 2-related factor 2 (NRF2) pathway, which regulates the expression of antioxidant genes and plays a protective role against oxidative damage [103]. Hyperglycemia-induced ROS overload disproportionately damages pancreatic β-cells, which have limited antioxidant defense. NRF2, a key transcription factor regulating antioxidant genes like glutathione S-transferase and NAD(P)H:quinone oxidoreductase 1, mitigates oxidative stress. Under basal conditions, NRF2 is suppressed by Keap1 proteins, but oxidants modify Keap1 cysteine residues, enabling NRF2 activation and transcription via antioxidant response elements (AREs) [103]. In T2DM, NRF2 expression is often reduced, leading to a diminished antioxidant response and the exacerbation of oxidative stress. However, NRF2 activation has been shown to improve insulin sensitivity, enhance insulin secretion, reduce vascular inflammation, and protect against diabetic complications, including CVD, albuminuria, and neuropathy [103,104,105,106,107]. Furthermore, NRF2 upregulation can suppress AGE-induced signaling through the RAGE, providing an additional mechanism to reduce oxidative stress and vascular damage in T2DM, as shown in both animal models and clinical studies [104,105,108].

The catabolism of glucose and lipids produces NADH and FADH2, which drive ATP synthesis via oxidative phosphorylation. However, T2DM-induced hyperglycemia and hyperlipidemia overwhelm this process, leading to excessive ROS production, mitochondrial dysfunction, and energy failure. This cascade contributes to cardiac hypertrophy, heart failure, and diabetic cardiomyopathy [109,110]. ROS-induced oxidative damage affects DNA, proteins, and lipids, ultimately triggering apoptosis and fibrosis. ROS also suppresses the PI3K/Akt/mTOR signaling pathway, which otherwise mitigates cell death. This imbalance promotes cardiac remodeling and dysfunction through fibrosis and metalloproteinase activation [111]. AGEs contribute to oxidative stress by interacting with RAGE, which activates downstream signaling pathways, including NOX and NFκB, further promoting inflammation and vascular damage. AGEs also cause stiffening of the vasculature, leading to increased blood pressure and cardiac fibrosis, which are key features of diabetic heart disease [108]. Furthermore, ROS-induced mitochondrial dysfunction and energy depletion contribute to the development of diabetic cardiomyopathy, a condition characterized by cardiac remodeling, fibrosis, and heart failure in T2DM [109,110]. ROS also suppress the PI3K/Akt/mTOR signaling pathway, which regulates cell survival and tissue repair, further promoting cardiac dysfunction and remodeling in diabetic patients [112].

Through these interconnected pathways, oxidative stress plays a central role in the pathogenesis of CVD in T2DM, contributing to endothelial dysfunction, atherosclerosis, cardiac remodeling, and heart failure. Addressing oxidative stress through targeted interventions could offer therapeutic potential for preventing or mitigating these complications in patients with T2DM.

## 4. Oxidative Stress and Cardiovascular Complications in T2DM

**Figure 2:**
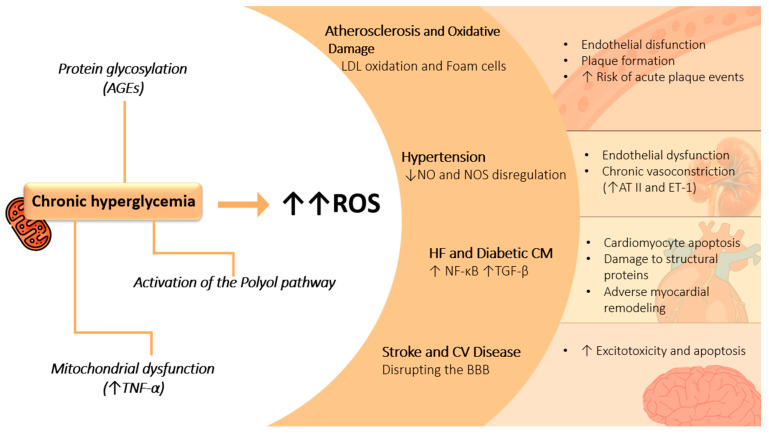
The figure illustrates the impact of chronic hyperglycemia on various cellular and systemic processes, leading to increased oxidative stress (ROS) and associated complications. Chronic hyperglycemia promotes the formation of advanced glycation end products (AGEs) through protein glycosylation, contributing to atherosclerosis and oxidative damage, which result in LDL oxidation and foam cell formation. It also activates the polyol pathway and induces mitochondrial dysfunction, increasing the production of ROS and elevating tumor necrosis factor alpha (TNF-α), which further amplifies oxidative stress. This cascade of events leads to several adverse outcomes, including endothelial dysfunction, plaque formation, and an increased risk of acute plaque events in atherosclerosis. In addition, chronic hyperglycemia contributes to hypertension by reducing nitric oxide (NO) availability and dysregulating nitric oxide synthase (NOS), causing chronic vasoconstriction through the elevated expression of angiotensin II (AT II) and endothelin-1 (ET-1). Furthermore, ROS exacerbates heart failure and diabetic cardiomyopathy by upregulating pro-inflammatory pathways like NF-κB and TGF-β, promoting cardiomyocyte apoptosis, damaging structural proteins, and inducing adverse myocardial remodeling. Lastly, ROS disrupts the blood–brain barrier (BBB), leading to excitotoxicity, apoptosis, and an increased risk of stroke and cardiovascular disease.

Cardiovascular complications in T2DM are diverse and include atherosclerosis, hypertension, heart failure, and stroke, all of which are significantly influenced by oxidative stress [113,114]. As previously discussed, chronic hyperglycemia in diabetes accelerates oxidative stress through mechanisms such as AGEs, the activation of the polyol pathway, and mitochondrial dysfunction. These pathways collectively create a heightened oxidative burden that significantly affects cardiovascular health (Figure 2) [115].

### 4.1. Atherosclerosis and Oxidative Damage

Atherosclerosis is a prominent cardiovascular complication in T2DM, independent of gender [116,117,118], and oxidative stress plays a fundamental role in its pathogenesis [119,120]. ROS actively oxidize LDL, forming oxLDL, a key event in atherogenesis [121,122]. OxLDL promotes macrophage uptake, transforming them into foam cells and initiating plaque development within arterial walls [123]. This process is exacerbated by hyperglycemia-induced ROS, which worsen endothelial dysfunction and create a pro-inflammatory milieu, further accelerating atherosclerotic plaque formation [121].

Oxidative stress not only initiates but also advances atherosclerotic plaques, rendering them unstable and prone to rupture, significantly increasing the risk of acute events such as myocardial infarction and ischemic stroke [124]. Elevated oxLDL levels in T2DM compromise plaque stability by promoting inflammation and extracellular matrix degradation, making plaques more vulnerable to rupture [125]. This underscores oxidative stress as a major contributor to cardiovascular morbidity in diabetes.

### 4.2. Hypertension

Hypertension, a common comorbidity in T2DM, is closely linked to oxidative stress, which depletes NO levels, a key regulator of vascular tone and endothelial function [126,127]. ROS react with NO to form peroxynitrite, reducing NO availability and impairing vasodilation. This leads to endothelial dysfunction and chronic vasoconstriction, perpetuating hypertension in diabetic patients [128,129].

ROS also disrupt vascular tone by influencing vascular smooth muscle cells (VSMCs). Oxidative stress activates the redox-sensitive signaling pathways that upregulate vasoconstrictive mediators such as angiotensin II and endothelin-1, resulting in increased vascular resistance [130,131]. This dysregulation, combined with inflammation, underscores the critical need for strategies to manage oxidative stress and its impact on hypertension in T2DM.

### 4.3. Heart Failure and Diabetic Cardiomyopathy

Oxidative stress is a significant contributor to the development of diabetic cardiomyopathy and heart failure in T2DM [132,133]. Excess ROS cause cardiomyocyte apoptosis and damage structural proteins, impairing myocardial contractility and leading to cardiac dysfunction [134]. Mitochondrial dysfunction further exacerbates oxidative stress, perpetuating cycles of cellular injury and adverse myocardial remodeling, including fibrosis and hypertrophy [135,136,137].

Key molecular pathways activated by oxidative stress in diabetic cardiomyopathy include NF-κB and transforming growth factor-beta (TGF-β), which drive inflammation and fibrotic remodeling [138]. Additionally, oxidative stress disrupts calcium homeostasis in cardiomyocytes, impairing contractility and exacerbating heart failure [133,139,140]. Targeting these mechanisms may offer therapeutic benefits in preserving cardiac function in T2DM.

### 4.4. Stroke and Cerebrovascular Disease

Stroke risk is markedly increased in T2DM, and oxidative stress plays a key role in exacerbating ischemic brain injury. ROS disrupt the blood–brain barrier, initiate inflammatory responses, and amplify neuronal damage during ischemia [141]. Furthermore, oxidative stress aggravates excitotoxicity and apoptosis, leading to more severe brain injury and poorer recovery outcomes in diabetic patients [142]. Antioxidant therapies could potentially mitigate these effects and enhance neuroprotection.

## 5. Lifestyle Modifications

### 5.1. Dietary Approaches

Dietary interventions play a critical role in managing oxidative stress and mitigating its impact on T2DM [143,144]. Diets rich in antioxidants and anti-inflammatory components can effectively reduce ROS production and improve insulin sensitivity, thereby addressing the metabolic disturbances underlying T2DM [145,146]. Among various dietary patterns, the Mediterranean diet, plant-based diets, low-glycemic index (GI) diets, and dietary approaches to stop hypertension (DASH) have garnered significant attention for their beneficial effects on oxidative stress, glycemic control, and cardiovascular health (Table 1) [147,148].

#### 5.1.1. The Mediterranean Diet

The Mediterranean diet, rich in antioxidants, has been widely associated with a reduction in oxidative stress. This diet emphasizes vegetables, legumes, fresh fruits, and whole-grain products (such as pasta and bread made with all types of grains). Cold-pressed extra-virgin olive oil (EVOO), nuts, and seeds are the primary fat sources, complemented by low dairy intake (two to three times per week), moderate fish, poultry, and egg consumption, minimal red meat (once a week), and moderate wine consumption with meals [149].

Polyphenols are the principal antioxidants in the Mediterranean diet, characterized by an aromatic ring with a hydroxyl group. These compounds are categorized into flavonoids and non-flavonoids, with hydroxycinnamic acids, flavonoids (e.g., quercetin and catechins), resveratrol, oleuropein, and hydroxytyrosol being the most studied [150]. Polyphenols are found in EVOO, whole grains, fruits, vegetables, nuts, tea, coffee, and red wine [151,152,153].

Flavonoids stimulate adiponectin secretion and AMPK phosphorylation, inhibiting NF-κB activation, inducible nitric oxide synthase (iNOS) pathways, and macrophage infiltration [154,155,156]. Hydroxytyrosol and oleuropein inhibit low-density lipoprotein oxidation [157,158]. Resveratrol reduces NADPH-induced oxidative stress and upregulates antioxidant enzymes like glutathione peroxidase and superoxide dismutase [159,160]. EVOO-derived polyphenols inhibit pro-inflammatory pathways (e.g., NOX-2, NOX-4) and cytokine expression (e.g., IL-1β, COX-2) while promoting PPARγ expression [161].

The Mediterranean diet also provides beneficial fatty acids, such as omega-9 (e.g., oleic acid from olive oil) and omega-3 (e.g., from fish). These fatty acids exhibit antioxidant and anti-inflammatory effects, improving pancreatic beta-cell function, insulin sensitivity, and endothelial health. Oleic acid decreases LDL levels, increases HDL levels, reduces ghrelin secretion, inhibits platelet aggregation, and may support hypothalamic regulation [162].

Thus, the Mediterranean diet plays a significant role in preventing and combating both metabolic and cardiovascular diseases [149,150].

#### 5.1.2. Plant-Based Diets

Research on plant-based diets and cardiovascular health has yielded mixed results. Studies such as AHS-2, ARIC, EPIC-Oxford, and the BROAD study have shown that vegetarianism is associated with reduced cardiovascular morbidity and mortality, improved BMI, cholesterol levels, and HbA1C. These effects may be linked to lower inflammatory mediator levels (e.g., CRP and white blood cells) and improved endothelial function [163,164,165,166]. Conversely, the EVADE-CAD study found no significant differences between vegan and AHA-recommended diets (rich in legumes, fruits, vegetables, nuts, grains, and fish) for controlling BMI, cholesterol, and HbA1C [163,167]. However, it reported reduced systemic inflammation (evidenced by lower hsCRP levels) in CAD patients following plant-based diets. Other studies have presented findings that contrast sharply with these results [163,164,165,166].

#### 5.1.3. Low-Glycemic Index Diets

Low-GI diets play a significant role in stabilizing blood glucose levels, which can reduce oxidative stress and improve insulin sensitivity in T2DM patients [168]. Foods with a low glycemic index, such as oats, legumes, and non-starchy vegetables, are slowly digested, leading to a gradual release of glucose into the bloodstream [169]. This slow release helps prevent the postprandial spikes in blood glucose that can trigger excessive ROS production and oxidative stress [170]. The reduction in ROS can alleviate inflammation and improve the function of antioxidant enzymes, such as superoxide dismutase and glutathione peroxidase, thus enhancing the body’s ability to manage oxidative stress effectively [171]. Additionally, research indicates that low-GI diets may reduce the risk of cardiovascular complications associated with T2DM by improving lipid profiles and endothelial function [172].

#### 5.1.4. DASH Diet

The DASH diet, originally developed to combat hypertension, has shown promise in managing oxidative stress and improving metabolic health in T2DM patients [173]. This diet emphasizes fruits, vegetables, whole grains, and low-fat dairy while reducing sodium intake [174]. It is designed to lower blood pressure, a common comorbidity in T2DM, and decrease oxidative stress markers [174,175]. Studies have demonstrated that adherence to the DASH diet results in improved endothelial function, reduced inflammation, and better regulation of blood glucose levels in T2DM patients [176]. Additionally, the diet’s rich antioxidant content—particularly from fruits and vegetables—helps mitigate oxidative damage by increasing the body’s intake of key nutrients like potassium, magnesium, and calcium, which are essential for cellular function and redox balance [177].

#### 5.1.5. The Japanese Diet

The Japanese diet, renowned for its health-promoting properties, offers a unique model for mitigating oxidative stress and reducing cardiovascular complications in T2DM [178]. Characterized by a high intake of fresh, minimally processed foods, the diet emphasizes fish, soy-based products, green tea, seaweed, vegetables, and whole grains, while maintaining low levels of red meat and saturated fats [178]. These dietary components are rich in bioactive compounds such as omega-3 fatty acids, isoflavones, catechins, and micronutrients, which collectively exert potent antioxidant effects [178]. Omega-3 fatty acids from fatty fish, for instance, have been shown to enhance endothelial function, reduce inflammation, and decrease ROS production, thereby protecting against vascular damage [179]. Isoflavones in soy foods upregulate endogenous antioxidant defenses and improve insulin sensitivity, while green tea catechins, particularly epigallocatechin gallate, directly scavenge free radicals and inhibit lipid peroxidation, contributing to improved glycemic control and vascular health [180]. Seaweed, a staple of Japanese cuisine, provides unique antioxidants such as fucoxanthin, which combat oxidative damage and support metabolic balance [181]. The diet’s low saturated fat content and emphasis on nutrient-dense foods further alleviate lipotoxicity and mitochondrial dysfunction, two key contributors to oxidative stress in T2DM [178,181]. Epidemiological evidence underscores the benefits of this dietary pattern, linking it to lower rates of cardiovascular disease and improved metabolic outcomes [182,183]. By addressing the oxidative stress pathways central to T2DM and its complications, the Japanese diet not only supports cardiovascular health, but also aligns with the broader goals of diabetes management, offering an effective strategy to reduce disease burden and improve quality of life [184].

#### 5.1.6. Ketogenic Diet

The ketogenic diet (KD), a high-fat, low-carbohydrate dietary regimen, has gained attention as a potential strategy for managing T2DM and mitigating associated cardiovascular complications [185]. By significantly reducing carbohydrate intake, KD induces a state of ketosis, wherein the body primarily uses fats for energy instead of glucose [186]. This metabolic shift has been shown to improve insulin sensitivity and reduce blood glucose levels, key factors in T2DM management [187]. Additionally, KD may help reduce oxidative stress, a major contributor to endothelial dysfunction and cardiovascular disease in T2DM patients. Some studies suggest that the KD lowers inflammatory markers and improves lipid profiles and mitochondrial respiratory complex activity, which can decrease the risk of cardiovascular complications [188]. However, the long-term effects of the ketogenic diet remain controversial, as it may have adverse effects on kidney function, lipid metabolism, and gut health if not properly managed [189]. More research is needed to fully understand the benefits and risks of the KD in T2DM, particularly in the context of oxidative stress and cardiovascular health. It is essential to consider patient-specific factors such as comorbidities, age, and adherence when recommending the ketogenic diet as part of a comprehensive approach to managing T2DM and preventing cardiovascular complications [190,191].

#### 5.1.7. Dietary Interventions and Gut Microbiota

Dietary interventions significantly influence gut microbiota composition, shaping its diversity and functionality, which in turn affects oxidative stress mechanisms in T2DM [192]. Diets rich in fiber, polyphenols, and unsaturated fats—such as the Mediterranean diet, plant-based diets, and the Japanese diet—promote the growth of beneficial gut microbes, including Bifidobacterium and Lactobacillus species [193,194,195]. These microbes produce short-chain fatty acids (SCFAs) like butyrate, which enhance intestinal barrier integrity, reduce systemic inflammation, improve glucose disposal and modulate oxidative stress by regulating NRF2 pathways and suppressing pro-inflammatory cytokines [196,197]. Conversely, high-fat or high-sugar diets foster dysbiosis, characterized by a decrease in microbial diversity and an overgrowth of harmful bacteria, which increase ROS production and exacerbate oxidative damage [198]. Emerging research highlights how microbial metabolites, such as SCFAs and tryptophan derivatives, interact with host metabolic pathways, directly influencing mitochondrial function and the redox balance [199,200]. These interactions not only mitigate oxidative stress but also improve insulin sensitivity and cardiovascular health.

### 5.2. Exercise

Physical activity is a cornerstone of lifestyle intervention in T2DM, exerting profound benefits through its ability to reduce oxidative stress, improve insulin sensitivity, and enhance vascular health [201]. Regular exercise stimulates adaptive responses in redox homeostasis, promoting endogenous antioxidant defenses while mitigating the inflammatory processes associated with metabolic dysregulation [202]. Different types of exercise, such as aerobic and resistance training, offer distinct yet complementary benefits in addressing oxidative stress and its downstream complications (Table 1) [203].

#### 5.2.1. Exercise as a Modulator of Oxidative Stress and Vascular Health

Regular physical activity, combined with a balanced diet and healthy lifestyle, significantly enhances overall health [204]. Exercise exerts beneficial effects by modulating oxidative stress and inflammation [205]. Chronic oxidative damage and inflammation contribute to cardiovascular complications such as endothelial dysfunction and arterial stiffness [206].

Studies demonstrate that aerobic exercise improves endothelial function by increasing nitric oxide bioavailability and reducing peroxide levels [207]. Long-term aerobic exercise induces shear stress-mediated arterial remodeling, enlarging artery sizes, reducing arterial wall thickness, and improving luminal reserve [208]. These adaptations decrease the risk of flow-limiting stenosis and arterial stiffness, particularly in individuals with metabolic syndrome or hypertension [208,209].

#### 5.2.2. Mechanisms of ROS Reduction and Enhanced Antioxidant Defenses

Exercise effects vary by type: endurance/aerobic vs. resistance training [210]. Endurance exercise temporarily increases O_2_^−^ production due to elevated metabolism, leading to ROS overproduction and oxidative stress [211]. Aerobic exercise can raise O_2_^−^ levels by one–three times during muscle contraction. However, mitochondrial O_2_^−^ generation decreases during exercise due to reduced mitochondrial NADH/NAD+ ratios, linked to a decline in complex I-dependent O_2_^−^ release [212]. During aerobic exercise, ATP breakdown produces ADP and, in some cases, AMP, which undergoes catabolism by xanthine oxidase (XO) to hypoxanthine, xanthine, and uric acid, further stimulating O_2_^−^ production and exacerbating oxidative stress [213]. Despite this, regular aerobic training boosts endogenous antioxidant enzyme activity, enhancing cellular capacity to mitigate ROS accumulation [202,211]. Moderate-intensity exercise conditions repair systems to combat oxidative damage and increase antioxidant defenses [213]. Conversely, insufficient physical activity reduces adaptive capacity in redox metabolism and homeostasis, underscoring the importance of regular exercise for oxidative stress management [213].

#### 5.2.3. Exercise-Induced Mitochondrial Biogenesis

Regular aerobic exercise plays a crucial role in enhancing mitochondrial function, an important mechanism for managing oxidative stress [214]. Exercise-induced mitochondrial biogenesis is regulated through the activation of key signaling molecules, such as peroxisome proliferator-activated receptor gamma coactivator 1-alpha (PGC-1α) and nuclear respiratory factor 1 (NRF1) [215,216]. These molecules promote the production of new mitochondria and enhance the efficiency of existing ones, leading to improved cellular energy metabolism [217]. The increased mitochondrial capacity allows cells to better handle oxidative stress by improving ATP production and reducing ROS generation [218]. Furthermore, enhanced mitochondrial function contributes to improved insulin sensitivity, which is critical for managing T2DM [219]. Long-term exercise also helps maintain redox balance by boosting antioxidant enzyme activity, thus protecting cells from oxidative damage [220].

#### 5.2.4. High-Intensity Interval Training

High-Intensity Interval Training (HIIT) is gaining recognition as a time-efficient exercise modality with significant benefits for T2DM management [221]. Unlike moderate-intensity continuous training, HIIT alternates between short bursts of intense activity and periods of low-intensity recovery [222]. This approach has been shown to improve maximal oxygen uptake (VO2 max), enhance insulin sensitivity, and reduce oxidative stress markers more effectively than traditional aerobic exercise [223]. The intense bursts of exercise during HIIT stimulate robust adaptations in muscle fibers, increasing their capacity for glucose uptake and improving mitochondrial function [224]. These effects not only help control blood glucose levels but also reduce ROS production during and after exercise [184]. Research suggests that HIIT can be particularly beneficial for patients with T2DM, as it offers a more efficient means of achieving metabolic improvements in a shorter amount of time [225].

#### 5.2.5. Exercise Synergy with Other Lifestyle Interventions

Combining regular exercise with dietary interventions, particularly the Mediterranean diet, can produce synergistic effects on oxidative stress management in T2DM [226]. Studies have shown that exercise and the Mediterranean diet together can amplify antioxidant enzyme activity, reduce systemic inflammation, and improve endothelial function more effectively than either intervention alone [227,228]. For example, the combination of aerobic exercise and a diet rich in polyphenols (such as those found in extra-virgin olive oil, fruits, and vegetables) has been shown to enhance the body’s defense against oxidative damage [229]. These lifestyle interventions work in tandem to improve glucose metabolism, reduce ROS production, and promote cardiovascular health, which are particularly important for patients managing T2DM [230]. This synergistic approach underscores the importance of a comprehensive lifestyle strategy in combating oxidative stress and metabolic disturbances associated with T2DM.

#### 5.2.6. Impact of Duration and Intensity of Lifestyle, and Individual Characteristics

The duration and intensity of dietary and exercise interventions are crucial in determining their effectiveness in managing oxidative stress and T2DM. Long-term adherence to dietary patterns such as the Mediterranean or DASH diets has been shown to provide significant metabolic and cardiovascular benefits, underlining the importance of sustained commitment over extended periods. These diets contribute to improved insulin sensitivity, reduced oxidative stress, and better overall metabolic control, particularly when maintained over months or years [231,232]. Similarly, exercise regimens that include moderate-to-high-intensity activities, such as at least 150 min per week of aerobic or combined aerobic and resistance training, have consistently demonstrated superior improvements in oxidative stress markers, insulin sensitivity, and cardiovascular health in individuals with T2DM [224]. The positive impact of exercise is enhanced when performed regularly and integrated with a balanced lifestyle, with evidence supporting its role in reducing the risk of complications associated with T2DM, such as cardiovascular disease [233]. However, the effectiveness of these interventions can vary significantly based on individual characteristics, including age, gender, race, and the stage of diabetes progression [234,235]. For example, older adults may face specific challenges such as decreased muscle mass, joint issues, or comorbidities like hypertension and cardiovascular disease, necessitating more tailored, gradual approaches to exercise [236]. In these individuals, the intensity and frequency of exercise may need to be adjusted to avoid injury while still providing sufficient benefits in terms of insulin sensitivity and oxidative stress reduction [237]. Furthermore, older adults often have a slower response to dietary and exercise interventions, and thus, a more prolonged intervention period may be required to achieve optimal metabolic outcomes [238]. Special consideration should be given to ensuring that exercise regimens are safe and accessible for this age group, with an emphasis on balance, flexibility, and functional strength, which are critical for improving overall health and reducing the risk of falls [239].

Gender differences also play a crucial role in the response to dietary and exercise interventions. Research has shown that women with T2DM may have a distinct metabolic profile compared to men, with different responses to exercise and dietary patterns [240]. For instance, women tend to have a higher percentage of body fat and may be more prone to insulin resistance, especially during menopause when hormonal changes further exacerbate metabolic dysfunction [241,242]. Therefore, exercise programs for women may benefit from a combination of aerobic and resistance training to increase lean muscle mass and improve insulin sensitivity [243]. Additionally, dietary interventions focusing on nutrient-dense, anti-inflammatory foods, as well as weight management, may be particularly beneficial in managing oxidative stress and improving insulin sensitivity in women [244].

Racial and ethnic disparities in the response to dietary and exercise interventions have also been well-documented, emphasizing the need for culturally sensitive approaches to managing T2DM [245]. Studies indicate that African American, Hispanic, and Asian populations often experience higher rates of T2DM and its complications compared to their white counterparts [246]. These differences may be attributed to a combination of genetic, environmental, and socioeconomic factors. For example, African American and Hispanic individuals tend to have higher levels of oxidative stress and inflammation, which may influence their response to dietary and exercise interventions [247,248]. Cultural dietary preferences, food availability, and lifestyle factors also play a role in the adherence and effectiveness of these interventions [249]. Tailoring dietary recommendations to suit cultural food preferences, as well as developing exercise programs that are accessible and relevant to these populations, can significantly enhance the effectiveness of lifestyle interventions [250].

The stage of diabetes progression further complicates the response to interventions. Individuals with early-stage T2DM may respond more rapidly and effectively to dietary and exercise interventions, showing significant improvements in insulin sensitivity and oxidative stress markers [251]. In contrast, those with advanced T2DM or long-standing poor glycemic control may require more intensive and individualized approaches, including the gradual intensification of exercise, medical management, and careful monitoring of comorbidities [252]. In such cases, interventions should be personalized to account for the individual’s specific health status, physical limitations, and risk of complications, ensuring that both short-term safety and long-term effectiveness are prioritized.

In light of these factors, personalized strategies that account for age, gender, racial differences, and the stage of diabetes progression are essential for optimizing the benefits of dietary and exercise interventions. Future research should focus on elucidating the complex interactions between these variables and their impact on intervention outcomes, with the goal of refining recommendations and enhancing their applicability across diverse populations.

**Table 1:** Effect of lifestyle intervention on oxidative stress in type 2 diabetes mellitus.

Category	Key Points	References
**Mediterranean Diet**	Rich in antioxidants from vegetables, legumes, fruits, whole grains, EVOO, nuts, and seeds.	[149]
	Polyphenols (e.g., hydroxycinnamic acids, quercetin, catechins, resveratrol, oleuropein, and hydroxytyrosol) are key antioxidants.	[149,150,151,152,153]
	Polyphenols enhance adiponectin secretion, AMPK activation, and inhibit NF-κB, iNOS, and macrophage infiltration.	[154,155,156]
	EVOO-derived compounds reduce LDL oxidation, NOX-mediated pathways, cytokines (e.g., IL-1β, COX-2), and increase PPARγ expression.	[157,158,161]
	Omega-3 and omega-9 fatty acids improve insulin sensitivity, beta-cell function, and endothelial health.	[162]
**Plant-Based Diets**	Shown to reduce cardiovascular morbidity/mortality, BMI, cholesterol, HbA1C, and inflammation in some studies (e.g., AHS-2, ARIC, EPIC-Oxford, BROAD).	[164,165,166]
	EVADE-CAD study found no significant advantages over AHA-recommended diets but noted reduced hsCRP in CAD patients.	[167]
**Low-Glycemic Index Diets**	Stabilizes blood glucose levels, reducing postprandial oxidative stress. Foods like oats, legumes, and non-starchy vegetables enhance antioxidant enzyme activities and reduce ROS production.	[168,169]
**DASH Diet**	Rich in fruits, vegetables, low-fat dairy, and low in sodium; improves blood pressure, reduces oxidative stress, and enhances endothelial function in T2DM patients.	[173]
	Increases antioxidant intake, reducing systemic inflammation and oxidative damage.	[173,174]
**Japanese Diet**	High in fish, seaweed, soy, and green tea; rich in antioxidants like catechins, isoflavones, and omega-3 fatty acids, which reduce oxidative stress and inflammation.	[178]
	Promotes improved endothelial function and reduced cardiovascular risk; omega-3 fatty acids support anti-inflammatory pathways.	[179]
	Fermented foods (e.g., miso, natto) may enhance gut microbiota and reduce systemic inflammation, contributing to oxidative stress modulation.	[193]
**Ketogenic Diet**	Induces ketosis by restricting carbohydrates and increasing fat intake, improving insulin sensitivity and reducing blood glucose levels.	[186]
	Lowers oxidative stress markers and inflammation, potentially improving endothelial function; effects on cardiovascular risk are still debated.	[188,189]
	May reduce mitochondrial ROS production but requires further long-term studies to assess safety and overall efficacy.	[188]
**Exercise Effects on Oxidative Stress**	Reduces oxidative stress and inflammation, improving endothelial function and vascular health.	[204,205,206]
**Aerobic Exercise**	Enhances nitric oxide bioavailability, reduces peroxide levels, and promotes arterial remodeling (e.g., larger arteries, thinner walls, reduced stiffness).	[207,208]
**Resistance vs. Aerobic**	Insufficient activity reduces redox adaptability, emphasizing the need for consistent exercise.	[212]

## 6. Future Directions

Future research should focus on deepening our understanding of the molecular mechanisms by which lifestyle interventions, such as diet and exercise, modulate oxidative stress in T2DM. The identification of key bioactive compounds in diets like the Mediterranean diet, such as polyphenols, and their interaction with metabolic pathways remains an exciting area for exploration. More personalized approaches that take into account genetic, environmental, and lifestyle factors will be essential for optimizing dietary recommendations and exercise regimens for individuals with T2DM [253]. In addition, the potential for combining lifestyle interventions with pharmacological therapies targeting oxidative stress pathways warrants further investigation. Advanced techniques such as metabolomics, nutrigenomics, and systems biology could provide invaluable insights into the complex interactions between diet, exercise, and oxidative stress [254]. Biobanks and longitudinal studies further enhance this potential by offering opportunities to analyze biological samples and track changes over time, facilitating the identification of long-term patterns and biomarkers associated with intervention efficacy. Research also needs to address the impacts of sex, age, and ethnic diversity in response to these interventions to ensure that treatment strategies are inclusive and applicable across different patient populations [255,256,257]. Moreover, integrating digital health technologies, such as wearable devices and mobile apps, to monitor oxidative stress biomarkers and lifestyle adherence could empower patients with real-time data, fostering sustained engagement and improving long-term health outcomes [258,259,260]. By addressing these gaps, future studies will enable the development of personalized, evidence-based strategies to mitigate oxidative stress and reduce cardiovascular risks in individuals with T2DM [261].

## 7. Conclusions

Oxidative stress plays a pivotal role in the pathophysiology of cardiovascular complications in T2DM. The imbalance between ROS production and antioxidant defenses drives endothelial dysfunction, vascular inflammation, and atherosclerosis, contributing significantly to the heightened risk of cardiovascular events in these patients. Key molecular pathways, including the activation of NOX and the dysregulation of NRF2, further exacerbate oxidative stress, promoting the development and progression of both microvascular and macrovascular complications. Addressing these mechanisms through lifestyle interventions such as dietary modifications and regular exercise presents a promising approach to managing oxidative stress and improving cardiovascular health in T2DM. The Mediterranean diet, rich in antioxidants, and plant-based diets have been shown to have beneficial effects on reducing oxidative stress, improving glycemic control, and mitigating cardiovascular risk factors. Similarly, exercise, particularly aerobic activity, plays a crucial role in enhancing antioxidant defenses, improving endothelial function, and reducing systemic inflammation. However, there remain many unanswered questions regarding the optimal intensity, duration, and combination of these interventions. Further research is needed to explore the synergistic effects of diet and exercise, as well as their molecular underpinnings, in the context of individual genetic profiles and disease progression.

## Data Availability

Not applicable.
